# Identification of Candidate Genes Associated with Trichothecene Biosynthesis in *Fusarium graminearum* Species Complex Combined with Transcriptomic and Proteomic Analysis

**DOI:** 10.3390/microorganisms10081479

**Published:** 2022-07-22

**Authors:** Jianhua Wang, Shanhai Lin, Guanghui Zhu, Zhiyong Zhao, Haoyu Wang, Miaoping Zhou, Xingming Zhao, Aibo Wu

**Affiliations:** 1 Institute for Agri-Food Standards and Testing Technology, Shanghai Academy of Agricultural Sciences, 1000 Jinqi Road, Shanghai 201403, China; wangjianhua@saas.sh.cn (J.W.); linshanhai@gxaas.net (S.L.); zhaozhiyong@saas.sh.cn (Z.Z.); m200300781@st.shou.edu.cn (H.W.); 2 Sugarcane Research Institute, Guangxi Academy of Agricultural Sciences, Nanning 530007, China; 3 Department of Computer Science and Technology, Tongji University, Shanghai 201804, China; moonshadowz@163.com; 4 Institute of Biotechnology, Jiangsu Academy of Agricultural Sciences, Nanjing 210014, China; mpzhou@jaas.ac.cn; 5 Institute of Science and Technology for Brain-Inspired Intelligence, Fudan University, Shanghai 200433, China; xmzhao@fudan.edu.cn; 6 SIBS-UGENT-SJTU Joint Laboratory of Mycotoxin Research, CAS Key Laboratory of Nutrition, Metabolism and Food Safety, Shanghai Institute of Nutrition and Health, University of Chinese Academy of Sciences, Shanghai 200031, China

**Keywords:** *Fusarium graminearum* species complex, trichothecene, mycotoxin, biosynthesis

## Abstract

The *Fusarium graminearum* species complex is the main causal agent of wheat head blight worldwide. Trichothecenes produced by the pathogen in infected grains have important food safety implications. Previously reported studies on trichothecene production have all focused on the conditions conducive to mycotoxin production, while the molecular mechanisms of trichothecene biosynthesis in *Fusarium* strains under normal or non-inducing conditions are still unclear. Here, a global analysis of the fungal gene expression of three strains using the Affymetrix *Fusarium* GeneChip under non-inducing conditions is reported. Differentially expressed genes were identified among strains with different trichothecene-production ability, and some novel genes associated with trichothecene biosynthesis were found by bioinformatics analysis. To verify the transcriptome results, proteomic analyses of the three strains were conducted under the same culture conditions. In total, 69 unique fungal proteins were identified in 77 protein spots. Combined with transcriptome and proteome analysis, 27 novel genes were predicted to be associated with trichothecene mycotoxin production. A protein, encoded by *FGSG_01403*, was found to be associated with trichothecene production via proteome analysis. Gene knock-out mutations of *FGSG_01403* resulted in mutants with increased production of trichothecenes. Future functional analysis of the candidate genes identified in this study may reveal new insights into the negative regulation of trichothecene production in the *Fusarium graminearum* species complex.

## 1. Introduction

The *Fusarium graminearum* species complex (FGSC), teleomorph *Gibberella zeae*, is an economically important plant pathogen that causes Fusarium head blight (FHB) in wheat, barley, and other cereals worldwide [1,2,3]. As one of the most destructive diseases in cereals worldwide, FHB epidemics in Europe and North America have resulted in huge economic losses [4]. Since 1990, the disease has caused over USD 3 billion in wheat and barley crop losses in the United States [5]. In China, FHB epidemics occur frequently along the middle and lower reaches of the Yangtze River, especially in Hubei, Anhui, and Jiangsu provinces, resulting in huge yield losses for farmers every year [6,7]. As recently reviewed by Chen et al. [8], from 2000 to 2018 the average yearly occurrence of FHB in China affected more than 4.5 million hectares, approximately 20% of the total planted area of wheat, and the annual yield losses were over 3.41 million tons. Today, it is clear that *F. graminearum* sensu stricto (s.s.) and *F. asiaticum* are the main causal agents of FHB in wheat in China [6,7,8,9,10].

Although yield losses caused by FHB are a major concern, significant levels of deleterious mycotoxins such as trichothecenes and zearalenones produced by the pathogens in infested grains pose a serious threat to human and animal health [1,11,12]. Fusarium toxins are generally stable and can be maintained during storage. Furthermore, these toxins can enter the food/feed chains during processing [13,14]. Fusarium toxins have been detected in different kinds of food products, such as bread, noodles, pancakes, malt, and beer [15]. Specifically, deoxynivalenol (DON) and its derivatives 3- and 15-acetyldeoxynivalenol (3ADON, 15ADON), trichothecene mycotoxins produced mainly by FGSC, are the common mycotoxins detected in cereal grains [16,17], with DON being the most abundant and more toxicologically significant to animals [18,19]. DON is a potent inhibitor of eukaryotic protein synthesis [20] and causes a variety of acute and chronic toxicoses when consumed by humans or animals [21,22]. Therefore, even low levels of mycotoxins in raw grain can make them unfit for human or animal consumption. Many countries regard DON and its derivatives as important regulatory targets, and the maximum residue limit of these compounds has been set in cereal grains. Furthermore, trichothecenes have previously been identified as virulence factors for fungi pathogenic to cereal crops [16,23,24], and the compounds have a significant role in determining the aggressiveness of the pathogen.

The trichothecene biosynthetic gene clusters and biosynthesis pathways in the FGSC and related species have been extensively studied in the past decade. As of now, 16 trichothecene biosynthesis genes (*TRI* genes), namely *TRI1*, *TRI3*, *TRI4*, *TRI5*, *TRI6*, *TRI7*, *TRI8*, *TRI9*, *TRI10*, *TRI11*, *TRI12*, *TRI13*, *TRI14*, *TRI15*, *TRI16*, and *TRI101*, have been characterized in the FGSC genome. Most of these *TRI* genes have been found located within a 25 kb region as a cluster in FGSC [25]. In addition to *TRI* genes that directly influence trichothecene production, many other genes have also been reported to be indirectly involved in trichothecene biosynthesis in the FGSC. These genes can interact with or regulate the expression of *TRI* genes (directly or indirectly) and ultimately influence trichothecene production. Functional genomics research results have indicated that some genes are essential for DON production in the FGSC. For example, *FgSho1* deletion mutants were blocked in DON production [26], while gene knock-out mutations of *FGSG_00007* and *FGSG_10397* resulted in mutants with a massively increased production of DON [27]. The protein kinase genes related to cyclic adenosine monophosphate (cAMP) signaling and three mitogen-activated protein (MAP) kinase pathways have been shown to be important for DON biosynthesis [28,29,30,31]. Thus, trichothecene biosynthesis is a complex secondary metabolic process, and a broad range of genes associate with its production need to be identified and characterized in the FGSC. The identification and characterization of novel genes or mechanisms involved in the regulation of trichothecene biosynthesis will extend our understanding of mycotoxin production and provide us with new insights into the development of comprehensive and effective disease and mycotoxin management.

The FGSC is among the most intensively studied fungal pathogens. With the availability of the full genomic sequence and annotation of the pathogen [32,33], a more in-depth analysis of mycotoxin production variation using a candidate gene approach is now possible. The development of an Affymetrix GeneChip laid the foundations for a variety of studies exploring the transcriptome of this organism under a variety of experimental and environmental conditions [34]. Up to now, a relatively large set of transcriptomic and proteomic studies on the FGSC have been carried out, but most have focused on the identification of pathogenicity-associated genes, such as virulence proteins and effector-coding genes [35,36,37,38,39,40,41,42,43]. The effect of FGSC strains on trichothecene production diversity according to transcriptomic or proteomic approaches has seldomly been explored [44,45,46]. The reported transcriptomic and proteomic studies on trichothecene production have all focused on the conditions conducive to mycotoxin production [44,45,46], while the molecular mechanisms of trichothecene biosynthesis in FGSC strains under normal or non-inducing conditions are still unclear.

The objective of the present study was to identify differentially expressed genes in different *Fusarium* strains under non-inducing conditions in vitro, using microarray analysis. This approach will serve as the basis for a better understanding of the molecular mechanisms involved in mycotoxin synthesis. To confirm the GeneChip results, proteins with significant differences in abundance under the same conditions were selected and identified through MALDI-TOF-MS/MS analysis. The results indicated that there are many unidentified genes associated with mycotoxin production in the FGSC. Further investigation into the functions of the novel identified genes could prove critical to the development of comprehensive and effective disease and mycotoxin management strategies.

## 2. Materials and Methods

### 2.1. Fusarium Strains

Three FGSC strains were selected to account for species and chemotype diversity within the FGSC, as well as diverse origins: PH1 (chemotype 15ADON, isolated on corn from Michigan, USA); F1 (chemotype 3ADON, isolated on wheat from Hubei, China); and 5035 (chemotype 15ADON, isolated on wheat from Hubei, China). The strains were routinely maintained on potato dextrose agar (PDA) plates at 25 °C.

### 2.2. Trichothecene Characterization

#### 2.2.1. Trichothecene Production in Rice Culture

Trichothecene production in rice culture was measured by inoculating autoclaved rice with 5 × 10^4^ conidial spores and culturing at 28 °C for 21 days in 250 mL Erlenmeyer flasks. In brief, 20 g of rice kernels and 10 mL of deionized sterile water were added into 250 mL Erlenmeyer flasks and kept at room temperature for 2 h, then autoclaved twice (121 °C, 30 min). After cooling at room temperature, the rice kernels were agitated until fluffy using a sterile glass rod in a clean bench, and then each flask was inoculated with 200 μL of 5 × 10^4^ conidial spores. Five flasks were used for each strain. After 21 days of incubation in the dark, the rice grains were dried and ground to powder, then extracted for trichothecene mycotoxin analysis.

#### 2.2.2. Trichothecene Accumulation in Living Plants

To assay mycotoxin accumulation in living wheat heads and to further verify the mycotoxin production ability of the three strains, PH1, F1, and 5035 were used to artificially inoculate four wheat cultivars (Sumai3, Ningmai13, Yangmai15, and Yangmai16) with differing FHB resistance. Harvested wheat spikelets of each treatment were pooled together and mashed into powder. Mycotoxins were extracted with an acetonitrile/water (84:16, *v*/*v*) mixture. Fresh spores of PH1, F1, and 5035 were generated from plugs grown in CMC medium. Per liter, the medium contained 0.5 g NH_4_NO_3_, 0.5 g KH_2_PO_4_, 0.5 g MgSO_4_·7H_2_O, 0.5 g yeast extract, and 7.5 g carboxymethyl cellulose–Na salt. After 5 days at 25 °C with continuous shaking at 200 rpm, spores were harvested by centrifugation (5000× *g*, 5 min) and counted using a hemacytometer. Spores were resuspended to 10^5^ spores per mL in sterile water for plant infection assay. The wheat cultivars Sumai3, Ningmai13, Yangmai15, and Yangmai16 were grown on experimental farm fields, Nanjing, China. At mid-anthesis, each biological replicate of fifty wheat spikes were point inoculated by pipetting 10 μL of a 1 × 10^5^ conidial suspension inside the middle spikelet, as described in [9]. Inoculated wheat heads were covered with polyethylene bags for 48 h to ensure constant high humidity. Mock inoculation using distilled water was carried out in parallel. Wheat spikelets were collected for analysis after 15 days to determine the concentration of trichothecenes (DON, 3ADON, and 15ADON) present.

#### 2.2.3. Trichothecenes Assay by UPLC-MS/MS

All the prepared trichothecene samples were tested as described by Zhao [47]. A Waters Acquity UPLC system equipped with an AB Sciex 5500 triple-quadrupole mass spectrometer (Darmstadt, Germany) was used for the analysis of trichothecenes. Chromatographic separation was carried out using a Waters Acquity UPLC^®^ BEH C18 column (2.1 × 100 mm, 1.7 μm) (Waters Corporation, Milford, MA, USA).

### 2.3. Microarray Protocol and Analysis

#### 2.3.1. Sample Preparation and Hybridization

For mycelia preparation, 1 mL conidial suspension (1 × 10^4^ spores) was inoculated into 250 mL Erlenmeyer flask containing 100 mL potato dextrose broth (PDB) medium, as described by Li [48], and six biological replicates were used for each strain. Cultures were incubated in the dark, with 150 rpm shaking at 28 °C for 21 days. At the end of incubation, the medium was poured through a two-layer Miracloth (Merck, Darmstadt, Germany). The mycelia collected from every second flask were pooled together, then washed three times with sterile water and freeze dried.

Total RNA of harvested mycelia was extracted using TRIzol reagent (Life Technologies, Carlsbad, CA, USA) according to the manufacturer’s instructions. RNA quality was verified using a 2100 BioAaalyzer (Agilent Technologies, Santa Clara, CA, USA). Prior to labeling, qualified total RNA was purified by a RNeasy mini kit (Cat#74106, QIAGEN, Hilden, Germany) and a RNase-Free DNase Set (Cat#79254, QIAGEN, Germany), according to the manufacturer’s guidelines. RNA amplification, labeling, and purification were performed following the manufacturer’s instructions. For gene-expression hybridization, each slide was hybridized with about 1.65 μg Cy3-labeled cRNA in a Hybridization Oven (Cat#G2545A, Agilent Technologies, Santa Clara, CA, USA). After hybridization, slides were washed and then scanned by the Agilent Microarray Scanner (Cat#G2565CA, Agilent technologies, US) with default settings. Data were extracted with Feature Extraction software 10.7 (Agilent Technologies), and before analysis raw data were normalized with a quantile algorithm using Gene Spring Software 11.0 (Agilent Technologies). The normalized expressions were further transformed by log_2_ function.

#### 2.3.2. Identification of Differentially Expressed Genes (DEGs)

Differential expression analysis was performed using the R package [49]. DEGs included two parts. The first part consisted of fold-changed genes, which had expressions that were not only significantly different from the background signals in at least all samples of one strain but also above a fold change of two in two strains. The fold changes of genes in two strains were calculated as follows, where *e*1 and *e*2 are the geometric mean expression of the gene in the two strains [50]. The second part consisted of specifically expressed DEGs that were significantly different from the background in all samples of one strain and not significantly different in each sample of the other strain.

$$FC=\frac{\left|e1-e2\right|}{min(g1,g2)}+1$$

#### 2.3.3. Functional Enrichment Analysis

Functional enrichment analysis of DEGs was performed using OE cloud tools (https://cloud.oebiotech.com/task (accessed on 6 June 2021)). The enriched biological processes, molecular functions, cellular components, and KEGG pathways were identified by Fisher exact test, and the *p*-value threshold for significant enrichment was 0.05.

#### 2.3.4. Identification of Shortest Paths

The shortest paths were identified by Dijkstra’s algorithm in the PPI network [51]. If there was more than one equal-length shortest path connecting a DEG and a *TRI*-family gene pair, all these paths were considered as possible paths for communication between *TRI*-family genes and DEGs.

#### 2.3.5. Identification of Gene Clusters in Shortest Paths (SPGs)

The gene clusters in the sub-network including SPGs as nodes and PPIs in shortest paths as edges were identified by ClusterOne [52]. The clusters with *TRI*-family genes and DEGs comprising more than half of the genes were identified as candidate clusters.

### 2.4. Proteomic Analysis

Mycelia cultured under the same conditions as the microarray experiment were subjected to proteomic analysis. Protein samples were prepared as described in [44]. Briefly, freshly harvested mycelia were ground with a mortar and pestle in liquid nitrogen. Total protein was extracted with chilled precipitation solution (20% *w*/*v* trichloroacetic acid (TCA) and 1% *w*/*v* dithiothreitol in acetone). After overnight precipitation at −20 °C, the protein pellet was obtained by centrifuged at 10,000× *g* for 20 min at 4 °C and washed three times with ice-cold acetone. Resolubilization of the precipitated proteins was carried out as described in [44]. The Bradford protein assay was performed to determine the concentration of protein. Proteins were labeled with cyanine fluorescent dyes (CyDyes™, GE Healthcare, Chicago, IL, USA) following the manufacturer’s instructions. Two-dimensional (2D) electrophoresis, image acquisition, and protein identification were performed according to the method reported by [44].

All identified proteins were assessed for N-terminal SP using SignalP 5.0 (https://services.healthtech.dtu.dk/service.php?SignalP-5.0 (accessed on 5 September 2021)), and probable transmembrane domains were determined using the TMHMM 2.0 Servers (https://services.healthtech.dtu.dk/service.php?TMHMM-2.0 (accessed on 5 September 2021)). The SecretomeP 2.0 server was used for predictions of non-classical secreted proteins (mammalian, NN score > 0.6). The chromosome locations and encoded protein sizes of the genes were determined by searching in databases from the EnsemblFungi for *F. graminearum* (strain PH1) (http://fungi.ensembl.org/index.html (accessed on 6 September 2021)).

### 2.5. Functional Analysis of FGSG_01403

#### 2.5.1. Generation of the *FGSG_01403* Deletion Mutant

To generate the gene replacement construct for *FGSG_01403* with the split-marker approach, we first used two primer pairs, 01403-1F/01403-2R and 01403-3F/01403-4R, to amplify the upstream and downstream fragments of the *FGSG_01403* gene from the genomic DNA of wild-type strain PH1. The resulting PCR products were separated by agarose gel electrophoresis and purified, then connected with the hygromycin phosphotransferase (*hph*) cassette by overlapping PCR. The gene-knockout cassette, containing *hph* marker with 5′ and 3′ flanking regions of *FGSG_01403*, further generated with the nested primer pair 01403-NF/01403-NR, was transformed into protoplasts of PH1 as described by Hou [28]. Hygromycin-resistant transformants were separated onto PDA plates, screened by a series of PCRs with different primer pairs and confirmed by Southern blot analysis. Southern blots were carried out according to the protocol supplied with the DIG High Prime DNA labeling and detection starter kit (Roche, Mannheim, Germany). Genomic DNA of wild-type and mutant strains was digested with HindIII, then fractionated and transferred to a membrane and probed with a DIG-labeled fragment of *FGSG_01403* gene and *hph* gene, respectively. All the primers used are listed in Table S3.

#### 2.5.2. Quantitative Real-Time PCR (qRT-PCR) Assays of TRI Genes

RNA samples of the wild-type strain PH1 and *FGSG_01403* mutant were isolated with TRIzol reagent (Invitrogen, MA, USA) from hyphae harvested from 6-day-old LTB cultures, as described previously [53]. RNA samples were treated with RNase-free DNase, and cDNA was synthesized with a TransScriptFly First-Stand cDNA synthesis kit (AF301-02, Transgen Biotech, Beijing, China) according to manufacturer’s instructions. The relative expression level of fungal genes was assayed as described previously [53,54]. The expression of a housekeeping gene of the fungi, the *TUB2 β-tubulin* gene [54], was used as a reference for normalization of the data. The relative expression levels of each gene were calculated using the 2^−^^ΔΔCt^ method. Primer sequences are listed in Table S3.

## 3. Results

### 3.1. Trichothecene Production Analysis of FGSC Strains

**Figure 1:**
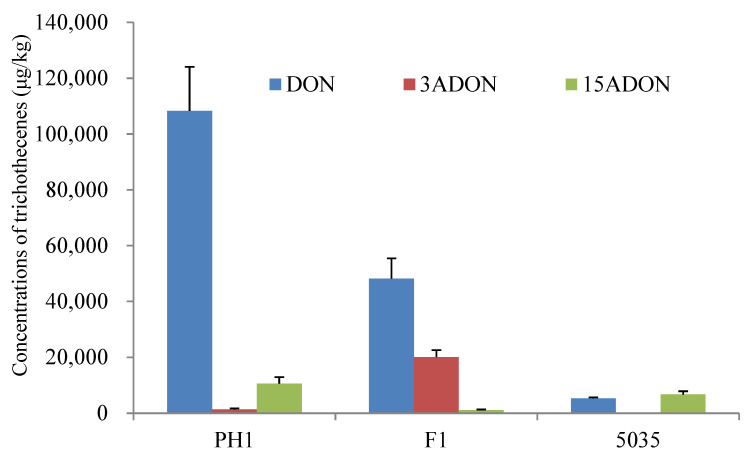
Chemotype and concentration determination of trichothecenes produced by the three *Fusarium* strains cultured in rice media.

Rice culture is widely used for trichothecene production assays in FGSC strains [7]. All the strains (PH1, F1, and 5035) could produce DON, 3ADON, and 15ADON in rice cultures in significantly varying amounts. As shown in Figure 1, strains PH1 and F1 produced more DON (2.41- to 80.99-fold) than 3ADON or 15ADON, whereas strain 5035 produced comparable levels of DON and 15ADON. For the acetylated DON, PH1 produced more 15ADON (7.89-fold) than 3ADON, F1 produced more 3ADON (17.67-fold) than 15ADON, and the amount of 15ADON produced by 5035 was 267.49 times higher than the amount of 3ADON. The results indicated that strains PH1 and 5035 were 15ADON producers, while strain F1 was a 3ADON chemotype.

The total amount of trichothecenes (including DON, 3ADON, and 15ADON) produced by PH1, F1, and 5035 in rice cultures was 120,190 μg/kg, 69,389 μg/kg, and 12,060 μg/kg, respectively. The concentration of total trichothecenes produced by F1 was 5.75 times higher than that produced by 5035. Among the three strains, the highest total amount of trichothecenes was produced by PH1 in rice media, which was 1.73-fold and 9.97-fold that produced by F1 and 5035, respectively. A representative LC-MS/MS chromatogram showing DON, 3ADON, and 15ADON is provided in Figure S1.

The trichothecene profile of each strain determined from artificially inoculated wheat heads, as shown in Figure S2, was identical to the results identified in rice cultures (Figure 1). On comparing the quantity of mycotoxins produced in living wheat heads by the three strains, the total amount of trichothecenes produced in the four wheat cultivars ranged from 23,929.36 to 38,765.70 μg/kg (average 30,275.31), 24,985.98 to 47,289.00 μg/kg (average 34,295.64), and 1993.99 to 13,149.68 μg/kg (average 7172.17) for PH1, F1, and 5035, respectively. Obviously, the total amount of trichothecenes produced by PH1 and F1 was much higher than that produced by 5035 in living wheat heads.

Based on the trichothecene production analysis in autoclaved rice cultures and living wheat heads, the three strains could be classified into different-chemotype and different-productivity groups, respectively. PH1 and 5035 are 15ADON chemotypes, while F1 belongs to the 3ADON chemotype, and PH1 and F1 are high trichothecene producers compared with 5035. In this study, strains PH1 and F1 were placed into the high-productivity group, while 5035 was placed into the relatively low-productivity group regarding trichothecene production.

### 3.2. Transcriptome Comparison of the Three FGSC Strains

The transcriptional profiling of the three *Fusarium* strains was carried out using a custom-designed Agilent oligomer array, which contained up to three individual 60-mers for each gene. Microarray analyses revealed that the transcriptomes of the three strains differed greatly. A total of 13,322 oligomers representing 13,322 fungal transcripts were perceived in the three strains. We detected 3552, 3315, and 3398 FGSC transcripts in PH1, F1, and 5035, respectively.

**Figure 2:**
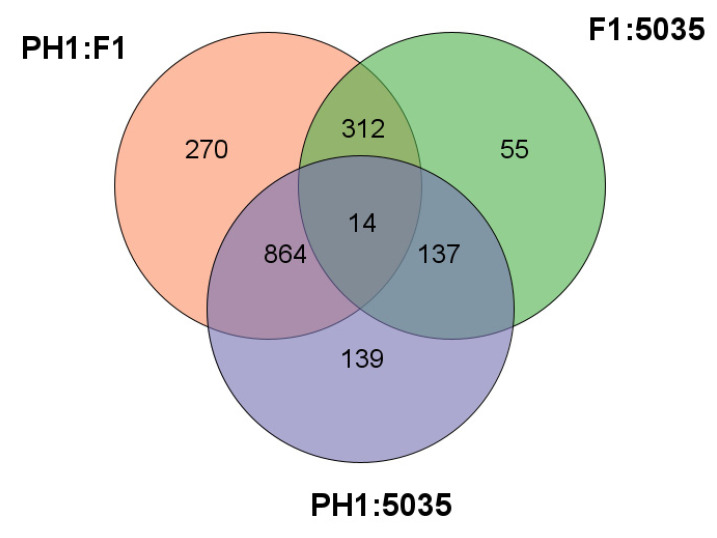
The intersections between DEGs identified through comparing each possible pair of F1, PH1, and 5035 strains are shown in Venn graph.

As shown in Figure 2, a total of 518 probe sets representing 518 genes in the PH1 annotation of the FGSC genome were significantly differentially expressed between PH1 and 5035, and 1154 probe sets representing 1154 genes were significantly differentially expressed between F1 and 5035. The expression profile of 3713 genes was evaluated between PH1 and F1, and 1460 (39.32%) genes were differentially expressed. Among the differentially expressed genes, 310 were up- or down-regulated (fold change, FC ≥ 2), and 1150 were specifically expressed in only one strain.

**Figure 3:**
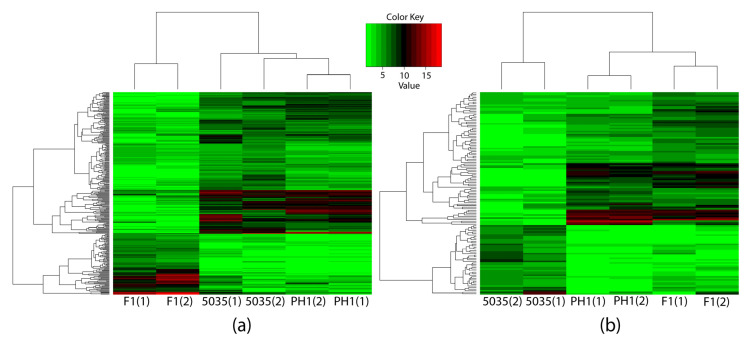
Three strains were clustered by expressions of identified DEGs through hierarchy clustering. The horizontal axis indicates the strains, and the numbers in parentheses represent different samples of the same strain. The vertical axis indicates the DEGs, the names of which are not shown in the figure. The expressions of the 296 DEGs identified through comparing different-productivity strains are shown in (**a**), while the 131 DEGs identified in the different-trichothecene-productivity group are shown in (**b**).

Specifically, to elucidate the mechanism of production and the chemotypes of trichothecene, we concentrated on comparing not only F1 with PH1 and 5035 but also 5035 with F1 and PH1. To identify the genes that caused the differences in the productivity and chemotypes of trichothecenes rather than the genes that only reflected the differences between strains, the DEGs between F1 and other strains (different-chemotype group) were restricted to genes not only differentially expressed between F1 and each of the other strains but also between F1 and the geometric mean expression of the other strains; this was the same when comparing 5035 with other strains (different-productivity group). Two hundred and ninety-six DEGs were detected in the different-chemotype group, with 131 DEGs in the different-productivity group. As shown in Figure 3, these DEGs distinguish the strain of the 3ADON chemotype and strains with high-productivity from strain 5035.

As previously reported in regard to trichothecene biosynthesis, *TRI* genes were specifically investigated across the three strains. In the different-chemotype group, the fold-changes of the expressions of *FGSG_00071* (*TRI1*) and *FGSG_03534* (*TRI3*) almost approached but were still lower than two, while their signals were not significantly different from the background in all samples of the F1 strain, and these two genes were not identified as DEGs. In the different-productivity group, there was no *TRI*-family gene identified as a DEG.

### 3.3. Annotation of Differentially Expressed Genes

**Figure 4:**
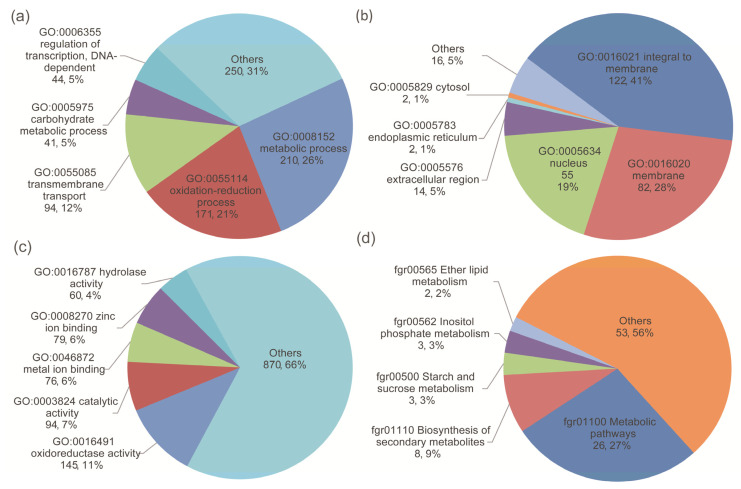
The DEGs were annotated with (**a**) biological processes, (**b**) cellular components, (**c**) molecular functions, and (**d**) KEGG pathways. The first and second numbers in each label are the number and percentage of DEGs annotated, respectively.

As there was no sufficient difference in the transcriptional level of the known trichothecene-related *TRI*-family genes, the functions of the DEGs in the two comparison groups were further investigated. The DEGs were annotated with gene ontology (GO) annotations from the Gene Ontology Annotation (GOA) database [50]. A total of 694 differentially expressed genes were assigned to GO categories (Figure 4). Among them, 131, 22, and 193 categories belonged to biological processes, cellular components, and molecular functions, respectively. The enriched GO terms in three two-strain comparison groups, the different-chemotype group, and the different-productivity group are listed in Table S1.

Out of 1791 non-overlapping differentially expressed genes, 34 were classified into 46 pathway categories from the KEGG database [55]. The most highly enriched pathway categories were metabolic pathways (76.47%), the biosynthesis of secondary metabolites (23.53%), starch and sucrose metabolism (8.82%), and inositol phosphate metabolism (8.82%), suggesting a high degree of basic metabolic activity in the regulation of gene expression under water stress conditions (Figure 4).

### 3.4. Comparative Proteome Analysis

**Figure 5:**
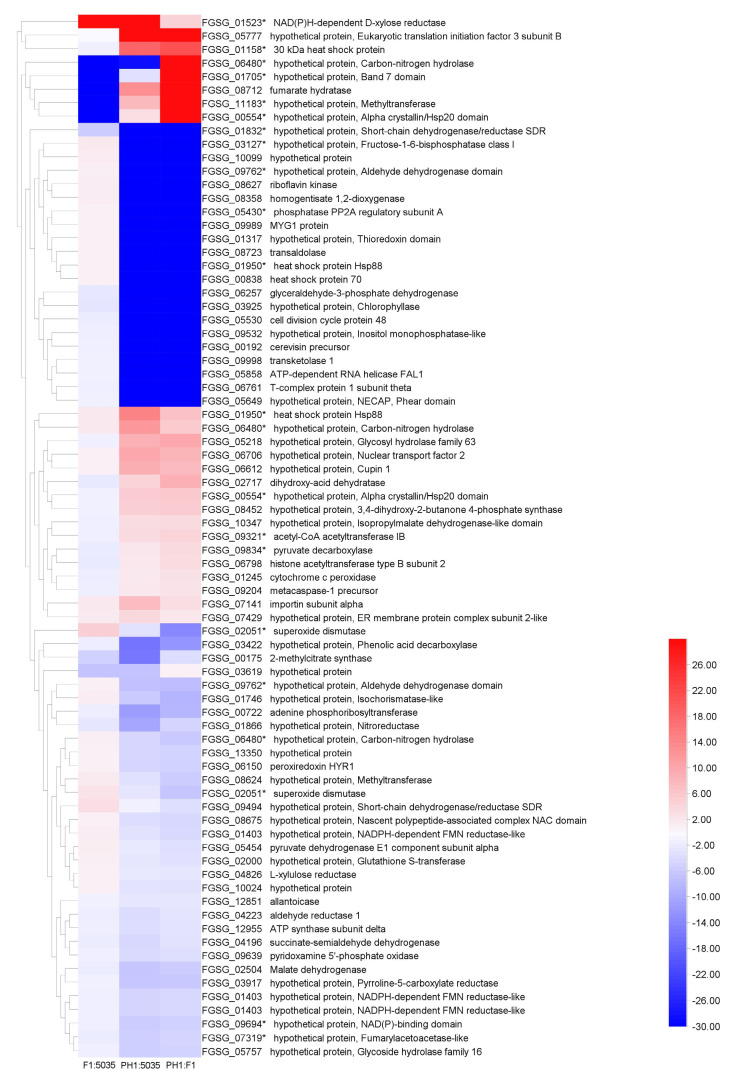
The heat map of 77 protein spots. Sixteen FGSG genes (identified in 22 protein spots) determined as DEGs in microarray analyses are indicated with asterisks. Blue represents lower expression; red represents higher expression.

By analyzing the protein profile of the three strains, we found that their overall protein patterns were quite similar but could still be discriminated. Protein spots with a significant increase (or decrease) in their relative abundance were considered to represent differentially expressed proteins. Proteome analysis focused on the abundance difference (*p* < 0.05, and more than a two-fold difference) between two of the three isolates, or the protein points that only appeared in one or two FGSC strains. Seventy-seven differentially expressed protein spots were trypsin-digested and subjected to MALDI-TOF-MS/MS analysis. Among these protein spots, 24 were significantly more highly expressed in PH1 compared with F1, 22 were more highly expressed in PH1 compared with 5035, and 7 were more highly expressed in F1 compared with 5035 (fold change > two). The heatmap of selected protein spots is shown in Figure 5. The FGSG accession numbers, protein sizes, annotations or potential functional domains, signal peptides (SP), and chromosome locations of the genes encoding the identified proteins are listed in Table S2.

The functions and locations of the identified proteins were analyzed according to the Uniprot Knowledgebase (Swiss-Prot/TrEMBL) and the Gene Ontology database. Approximately 13% of proteins were involved in the biosynthetic process; 13% were involved in the small molecule metabolic process; 23% were related to nitrogen or carbohydrate metabolism; and the rest of the proteins mostly displayed catalytic enzyme activity, transferase activity, and so on. It was found that the identified proteins were mainly involved in metabolism, structure, and signal transduction. The differential expressed proteins were located in the cytoplasm, nucleus, mitochondrion, protein complex, intracellular, cytosol, and organelle.

Presence of N-terminal SP sequences and the probable transmembrane regions of individual proteins were determined using the SignalP 5.0 and TMHMM 2.0 Servers based on the amino acid sequences, respectively. Among the 69 proteins, 4 (*FGSG_00192*, *FGSG_05218*, *FGSG_05757*, *FGSG_06612*) were predicted to contain an N-terminal SP, while no SP sequences were predicted in the other 65 proteins. No transmembrane regions were identified in the 4 N-terminal SP-containing proteins by TMHMM analysis, indicating that they are secreted proteins.

For the 65 N-terminal SP-lacking proteins, 2 (*FGSG_01245* and *FGSG_03917*) were predicted to contain at least one transmembrane region, and no transmembrane regions were found in the other 63 proteins. These 63 proteins containing neither SPs nor transmembrane regions were further subjected to SecretomeP predictions for non-classically secreted proteins. In total, 15 fungal proteins identified in 16 spots obtained an NN-score exceeding the threshold (mammalian, 0.6) predicted by SecretomeP, indicating that these may be secreted in non-classical pathways.

### 3.5. Linking of Differentially Expressed Genes, Proteins, and Module Snalysis

Based on the fact that interacting proteins rather than individual proteins tend to perform the functions of cells, the DEGs, whose roles in trichothecene production were unclear, and the *TRI*-family genes, which were previously reported to be involved in the process but were barely differentially expressed, should be jointly analyzed to elucidate the mechanism of trichothecene production. We utilized a predicted PPI network obtained from the EFG database [56] to predict how the DEGs interacted with the *TRIs* to participate in trichothecene production. For each possible *TRI* gene and DEG pair, we identified the shortest paths in the PPI network connecting them. Reasonably, the more times a gene was crossed through by these paths, the more likely its participation in the trichothecene production process.

**Figure 6:**
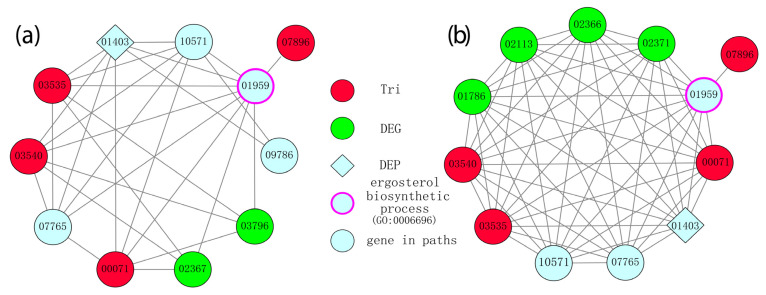
Gene clusters were identified in the sub-network consisting of the shortest paths connecting DEGs and *TRI*-family genes for (**a**) different-chemotype and (**b**) different-productivity groups. Nodes represent genes contained in the shortest paths, while edges represent the PPIs made up of paths. The *TRI*-family genes are marked as red, while the differentially expressed protein FGSG_01403 identified in proteome analysis and differentially expressed genes (DEGs) are marked as rhombi and green, respectively.

Eighty-six DEGs and twenty-eight DEGs were found in the different-chemotype and different-productivity groups, respectively, in the PPI network, out of which seventy and twenty-four DEGs were connected with four common *TRI*-family genes (*FGSG_00071* (*TRI1*), *FGSG_03535* (*TRI4*), *FGSG_03540* (*TRI11*), *FGSG_07896* (*TRI101*)). The biological processes in the genes comprising the shortest paths were identified to validate the association between these genes and the production of trichothecene (Figure 6). Compared to the DEGs, which were not significantly enriched with terms related with trichothecene production, a significant association between shortest-path genes (SPGs) and trichothecene production was observed. For example, the GO:0006696 ergosterol biosynthetic process (*p*-value 0.022, Fisher exact test) and GO:0009405 pathogenesis (*p*-value 0.022, Fisher exact test) were enriched in SPGs in the different-chemotype group, while the GO:0006696 ergosterol biosynthetic process (*p*-value 0.0035, Fisher exact test) was enriched in the different-productivity group. These enriched biological processes indicated that the identification of SPGs reliably revealed more genes in the process of trichothecene production than that of DEGs.

As dense structures in a PPI network represent co-operating proteins in biological functions, gene clusters in the sub-network consisting of SPGs were identified as candidate gene clusters participating in trichothecene production in the different-chemotype and different-productivity groups. As shown in Figure 6, the *TRI*-family genes, DEGs, differentially expressed proteins (DEPs), and gene *FGSG_07896* annotated with the GO:0006696 ergosterol biosynthetic process interacted with each other in a dense way, as they were crossed through by the shortest paths more times than the genes outside the clusters, indicating the importance of the two clusters in the process of trichothecene production. Similarly, the *TRI*-family genes, DEGs, DEPs, and other genes that were members of the clusters and closely interacted with the *TRI*-family genes were identified as novel genes related to trichothecene production.

Based on the module analysis (Figure 6), 11 candidate genes (*FGSG_01403*, *FGSG_10571*, *FGSG_01959*, *FGSG_09786*, *FGSG_03796*, *FGSG_02367*, *FGSG_07765*, *FGSG_01786*, *FGSG_02113*, *FGSG_02366*, and *FGSG_02371*) associated with trichothecene biosynthesis in FGSC strains were identified. These genes have not yet been assigned specific biological functions, so their precise activities remain unknown. The functional analysis of these genes will serve as the basis for a better understanding of the molecular mechanisms involved in mycotoxin synthesis in the fungus.

A combinatorial analysis of the transcriptomic and proteomic data was conducted, and overlapping differentially expressed genes and proteins were revealed. In total, 16 of 69 identified DEPs were differentially expressed according to microarray analysis (Figure 5 and Table S2). These DEPs may also be involved in trichothecene biosynthesis in the FGSC, and their functions should be further characterized. According to the results above, it is obvious that transcriptomic and proteomic approaches, and especially their combination, are powerful tools for monitoring or identifying the genes involved in trichothecene production in the FGSC under certain conditions.

### 3.6. FGSG_01403 Mutant Produced Higher Levels of Trichothecenes

To determine the role of *FGSG_01403* in the biology of the FGSC, the gene was knocked out by replacing it with a hygromycin-resistant cassette containing a *hph* marker with 5′ and 3′ flanking regions of *FGSG_01403*, using homologous recombination in the wild-type strain PH1. Hygromycin-resistant transformants were screened by a series of PCRs with different primer pair combinations, such as 01403-JDF1/R1, 01403-1F/HYR, and YGF/01403-4R (Table S3), and confirmed by Southern blot analysis (Figure S3). The trichothecene production of the mutant (fgsg01403) was determined in rice cultures after a 6-day incubation period, and four replicates were made for each strain. In comparison with the wild-type strain, the amount of trichothecenes produced by the *FGSG_01403* mutant (55,660 μg/kg) was 8.29-fold that produced by PH1 (6710 μg/kg) (Figure S4). No obvious effects on the growth of the fungus were observed.

The expression level of trichothecene biosynthetic genes in PH1 and the *FGSG_01403* mutant cultured in liquid trichothecene biosynthesis (LTB) medium was validated by qRT-PCR assays. When assayed with RNA isolated from hyphae collected from 6-day-old cultures, the expression levels of *TRI1*, *TRI3*, *TRI4*, *TRI5*, *TRI6*, *TRI10*, *TRI11*, and *TRI101* were more than two-fold higher in the *FGSG_01403* mutant than in the wild-type strain, while no significant difference was observed in the expression of *TRI8* between the wild-type strain and the mutant (Figure S5). Therefore, FGSG_01403 is a suppressor of trichothecene biosynthesis and negatively regulates *TRI* gene expression and mycotoxin production in the FGSC.

## 4. Discussion

A high level of molecular diversity has been reported in *Fusarium* populations worldwide. Genetic variation among isolates belonging to the FGSC is an extensively described fact. Variations in the culture characteristics, aggressiveness, and trichothecene (chemotype and concentration) production have been found among strains sampled in the same country or even the same field [3,44,57,58,59,60]. Goswami and Kistler [3] tested on a global scale the aggressiveness and trichothecene production of 31 strains belonging to eight species of FGSC and originating from diverse hosts or substrates. The results showed that there was significant variation among strains of the FGSC in their ability to produce trichothecenes on wheat, and they concluded that the variation appeared to be a strain-specific rather than species-specific characteristic [3]. Comparing 12 individual field populations each consisting of 30 strains, Talas et al. [59] reported that more than 70% of the molecular variation in aggressiveness and trichothecene production was identified within populations and less than 30% between populations. For our chemical analysis, we found that the most abundant trichothecenes were produced by strain PH1 under all conditions (autoclaved medium and living host tissues). A similar trend in trichothecene production was observed in different biological replicates under the same conditions. In addition, no significant difference was observed among different technical replicates. Consistent with the previous findings in [3,44,57,58,59,60,61,62], we concluded that the trichothecene production ability of FGSC strains is a strain-specific characteristic, and the high diversity among individual strains reflects the high evolutionary potential of FGSC strains. Trichothecene production is an important factor determining the potential contamination level of an isolate. Because of the critical role trichothecenes play in spreading disease and their potent threat posed to food safety, it is important to study the molecular mechanisms of trichothecene biosynthesis to gain a comprehensive understanding of the processes that may be useful for developing strategies to control pathogenic fungi and trichothecene biosynthesis.

The molecular causes of this tremendous variation among different strains are still unclear [60]. Many studies on trichothecene production have been carried out, but all have focused on single-strain analysis or on comparing mutants obtained from the same isolate under mycotoxin-inducing conditions [44,45,46]. To date, the molecular mechanisms of trichothecene biosynthesis in FGSC strains under normal or non-inducing conditions have not been reported. Omics data of FGSC strains obtained from trichothecene non-inducing conditions will allow the exploration of the genome diversity, RNA or DNA expression level differences, and protein or metabolic molecule abundance variation. These differences may reflect the genetic basis for mycotoxin biosynthesis and facilitate the process of inferring the general biological mechanisms involved in trichothecene production. Normally, the *TRI* clusters remain silent and are mainly controlled by repressors that negatively regulate trichothecene biosynthesis in the FGSC. The DEGs and DEPs identified in this study may have resulted from ecological adaptability and evolutionary selection pressure, which probably cannot be observed under trichothecene-inducing conditions. These variations can be accounted for by underlying genetic differences. There was no *TRI*-family gene identified as a DEG in our transcriptome analysis; the reason for this is the non-conducive media used in this study.

*Fusarium* strain diversity has been seldomly explored using proteomics [44], especially concerning genetic chemotype diversity or trichothecene production quantity diversity. A whole-cell 2D-DIGE proteomic analysis of 3ADON, 15ADON, and NIV genetic chemotype FGSC strains was performed by Pasquali et al. [44], and a strain-comparative proteomic approach was used to identify regulatory changes triggered by agmatine. The results indicated that agmatine augmented trichothecene production, but not equally across all strains. By using multiple strains, the shared mechanisms of regulation induced by agmatine were identified. Their findings demonstrated the usefulness of exploring strain omics diversity (genomic, transcriptomic, proteomic, metabonomic) within species not only to characterize the level of diversity among strains with different phenotypic manifestations but also to facilitate the process of inferring general biological mechanisms by identifying shared biological processes among the strains [44].

All 77 protein spots were successfully identified, and these spots matched well with 69 proteins of the *Fusarium* strain (PH1), which corresponded to 69 unique FGSG numbers. Thirty-four (49.28%) of these sixty-nine proteins were uncharacterized or hypothetical proteins. Seven proteins (*FGSG_01403*, *FGSG_01866*, *FGSG_02051*, *FGSG_04826*, *FGSG_06257*, *FGSG_08723*, and *FGSG_09834*) have been reported previously in proteomics studies of the FGSC [41,46,63]. Protein isoforms were detected for six FGSG numbers (*FGSG_00554*, *FGSG_01403*, *FGSG_01950*, *FGSG_02051*, *FGSG_06480*, and *FGSG_09762*) among these sixty-nine proteins. Two of these fungal proteins (*FGSG_00554*, *FGSG_01403*) were present in multiple spots with distinct *p* values, indicating either post-translational modification events (acetylation, phosphorylation, etc.) occurring in the same gene product or the existence of closely related gene products arising from alternative splicing, RNA editing [64], etc. Compared with the microarray data, we found that 16 proteins (Figure 5) were determined as DEGs in transcriptomic analysis.

Non-classical secretory proteins have previously been reported from the secretome analysis of FGSC strains. The secretome of *F. graminearum* strain PH1 grown with barley or wheat flour as the sole nutrient source were analyzed by a gel-based proteomics approach [41]. Among the 69 unique fungal proteins identified, 11 (16%) were predicted to be secreted in a non-classical manner [41]. In our study, a moderately higher proportion (almost 22%) of non-classical secretory proteins were identified. In the study by Paper et al. [40], an even higher proportion (around 56%) of proteins lacking SPs was observed in extracellular extracts of FGSC-infected host tissues. Similarly, proteins secreted by a nonclassical means in other fungi, e.g., *Aspergillus fumigates*, *Candida albicans*, *Claviceps purpurea*, and *Saccharomyces cerevisiae*, have been reported previously [41,65].

We noticed that FGSG_01403 was highly expressed both in F1 and 5035 compared with PH1, while the amount of mycotoxin produced by PH1 was 3.47- and 21.38-fold that produced by F1 and 5035, respectively. Bioinformatics analysis indicated that the *FGSG_01403* gene was 848 bp in length with a 233 bp intron, and the gene encodes a hypothetical protein with 204 amino acids in the FGSC; its function has not been identified before. *FGSG_01403* is located on *F. graminearum* chromosome 1, and thus is not co-located with any core *TRI* gene involved in trichothecene metabolism. The promoter region of *FGSG_01403* was searched for the presence of putative TRI6-binding sites [66]; surprisingly, a GTGA/TCAC motif separated by seven nucleotides was identified in the promoter region (-1212 to -1198). In total, ten individual GTGA/TCAC TRI6 binding sites were found in the promoter region of *FGSG_01403*. Gene disruption indicated that FGSG_01403 appears to have a role in capping the production of trichothecenes, because the mutant produced significantly greater quantities of trichothecenes than the wild-type strain. The expression levels of all the assayed *TRI* genes, expect for *TRI8*, were significantly increased in the *FGSG_01403* mutant compared with the wild-type according to qRT-PCR analysis. This is consistent with the fact that the *TRI8* gene is constitutively expressed when the fungus is grown on both trichothecene-producing and non-producing media [25].

To date, a limited number of genes negatively regulating trichothecene production have been identified in the FGSC genome. *FGSG_00007* and *FGSG_10397* were identified in a gene expression profile of the FGSC in a *TRI6* mutant by microarray analyses, and the quantification of trichothecenes from their deletion mutants showed large increases in the amounts of mycotoxins [27]. The function analysis of the two phosphodiesterase genes, *PDE1* and *PDE2*, showed that the deletion of *PDE2* increased trichothecene production in the FGSC [67]. Further studies on the novel mechanism of the negative regulation of trichothecene production in the FGSC are needed. The identification of the negative regulation mechanism of trichothecene production in the FGSC also points out the potential of this pathogen to evolve with an ability to produce massively increased amounts of mycotoxins [27].

It is known that trichothecenes are not essential for survival but instead enable a pathogen to successfully infect its plant hosts. Given the high functional specificity and energetic cost, normally, the *TRI* clusters remain silent unless the organism is subjected to an environment conducive to trichothecene biosynthesis. *TRI* gene clusters can be activated by genetically manipulating their activators or repressors. Under inducing conditions, effectors involved in trichothecene production will be triggered, while the suppressors may be suppressed. On the contrary, under non-inducing conditions, the *TRI* gene clusters will be mainly controlled by the repressors that negatively regulate mycotoxin production in the FGSC. In other words, conditions conducive to trichothecene production will facilitate the detection of trichothecene activators in the FGSC. On the other hand, negative regulators would most probably be easier to identify under non-inducing conditions, like the experiments designed in this study. The fact that novel genes associated with trichothecene production have been identified under different conditions suggests that the biosynthesis of mycotoxins is a complicated process and regulated by many factors in the FGSC, and the general biological mechanisms are yet to be fully determined.

## 5. Conclusions

In this study, we performed a comparison of the transcriptome of two high-level mycotoxin producers (PH1, F1) and a relatively low-level mycotoxin producer (5035). Our results showed strong similarity between the transcriptomes observed in PH1 and F1. In total, 1154 DEGs were identified between PH1 and 5035, and 245 of them were connected to *TRI* genes. Additionally, 518 DEGs were identified between F1 and 5035, and 120 of them were connected to *TRI* genes. The linkages between known trichothecene biosynthesis genes and differentially expressed genes were assayed, and several novel genes associated with *TRI* genes were found. Based on mold analysis, 11 genes were identified: *FGSG_01403*, *FGSG_10571*, *FGSG_01959*, *FGSG_09786*, *FGSG_03796*, *FGSG_02367*, *FGSG_07765*, *FGSG_01786*, *FGSG_02113*, *FGSG_02366*, and *FGSG_02371*. Most of these genes were involved in arginine and proline metabolism, the MAPK signaling pathway, the cAMP pathway, and the arginine metabolic process. Furthermore, comparative proteome analysis was conducted by 2D electrophoresis, and 77 differentially protein spots were subjected to MALDI-TOF-MS/MS analysis. These proteins matched well with 69 proteins of the FGSC, and one of these proteins (*FGSG_01403*) was highly expressed both in F1 and 5035 compared with PH1. Gene disruption indicated that *FGSG_01403* appears to have a role in capping the production of trichothecenes, because the mutant produced significantly higher quantities of trichothecenes than the wild-type strain. Combined with proteome analysis and microarray analysis, another 16 DEPs were identified in GenChip. This study revealed a suite of novel genes (27 genes, Table S4) associated with trichothecene production in the FGSC. Future functional analysis of the genes identified here may reveal new insights into the regulation of trichothecene production in this economically important fungal pathogen.

## Data Availability

The data presented in this study are available from the corresponding author upon reasonable request.

## Supplementary Materials

The following supporting information can be downloaded at: https://www.mdpi.com/article/10.3390/microorganisms10081479/s1, Figure S1: Representative LC-MS/MS chromatograms for DON, 3ADON, and 15ADON, Figure S2: Trichothecenes produced in wheat heads on wheat cultivars Sumai3, Ningmai13, Yangmai15, and Yangmai16, Figure S3: PCR identification and Southern blot analysis of *FGSG_01403* deletion mutants, Figure S4: Trichothecene production of PH1 and *FGSG_01403* mutant fgsg01403 in 6-day-old rice cultures, Figure S5: The expression levels of *TRI* genes in the wild-type PH1 and *FGSG_01403* mutant fgsg01403, Table S1: The enriched GO terms in different comparison groups, Table S2: Differentially accumulated protein spots identified in this study from the mycelial proteomes of *Fusarium* strains, Table S3: Primers used in this study, Table S4: List of genes associate with trichothecene production identified in this study.
