# Supplementary material for: Identification of Candidate Genes Associated with Trichothecene Biosynthesis in Fusarium graminearum Species Complex Combined with Transcriptomic and Proteomic Analysis
Source: Microorganisms. 2022 Jul 22;10(8):1479. doi: 10.3390/microorganisms10081479 (PMC9332169; doi:10.3390/microorganisms10081479)
Supplement: Supplementary file 1 [file microorganisms-10-01479-s001.zip › Figure S2.pdf]

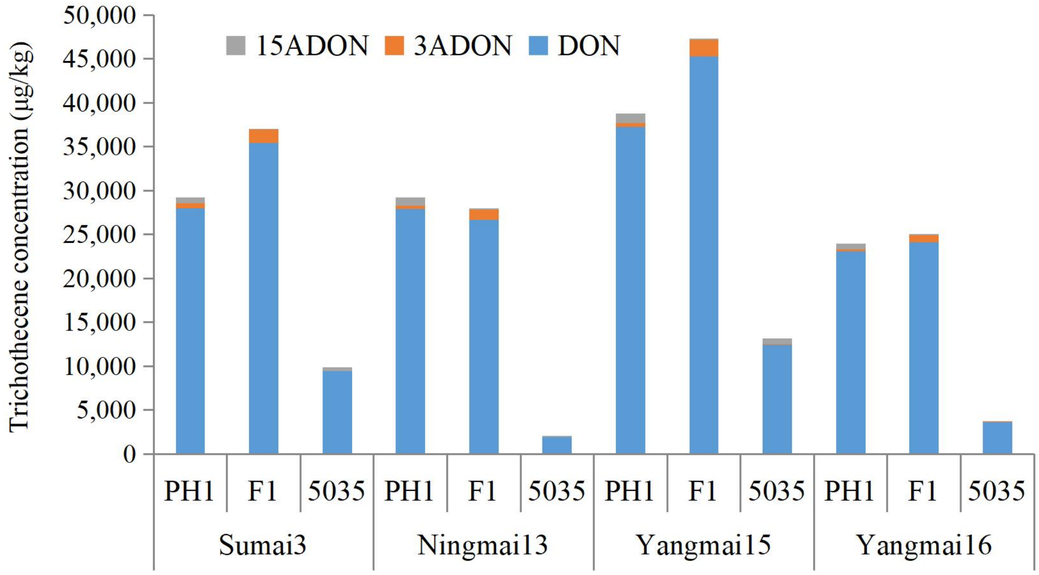

Figure S2. Trichothecenes produced in wheat heads on wheat cultivars Sumai3, Ningmai13, Yangmai15, and Yangmai16.
