# Supplementary material for: Identification of Candidate Genes Associated with Trichothecene Biosynthesis in Fusarium graminearum Species Complex Combined with Transcriptomic and Proteomic Analysis
Source: Microorganisms. 2022 Jul 22;10(8):1479. doi: 10.3390/microorganisms10081479 (PMC9332169; doi:10.3390/microorganisms10081479)
Supplement: Supplementary file 1 [file microorganisms-10-01479-s001.zip › Figure S3.pdf]

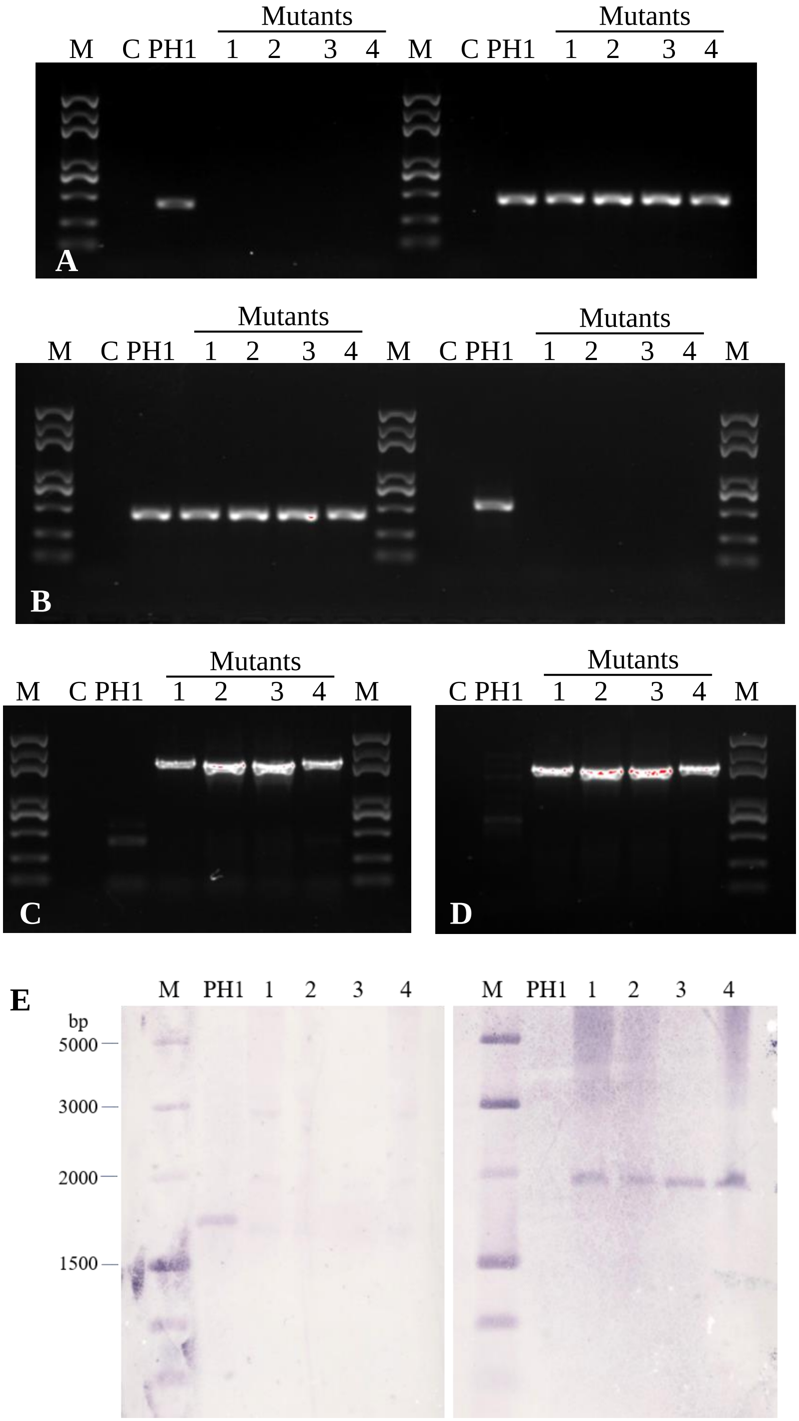

Figure S3. PCR identification and Southern blot analysis of *FGSG_01403* deletion mutants.

(**A**) Amplified with primer pair 01403-JDF1/01403-JDR1 (left) and primer pair Fg16F/Fg16R (right).

(**B**) Amplified with primer pair TEF-F/TEF-R (left) and primer pair 01403-JDF2/01403-JDR2 (right).

(**C**) Amplified with primer pair 01403-1F/HYR.

(**D**) Amplified with primer pair YGF/01403-4R.

(**E**) Southern blots analysis of transformants using the *FGSG_01403* gene (left) and *hph* gene (right) as probes, respectively.

Codes numbers above the panel correspond to the wild-type strain PH1 and *FGSG_01403* deletion mutant codes. Lane M, DNA marker; Lane C, negative control (omitting DNA template).
