# Supplementary figures and images for: Identification of Candidate Genes Associated with Trichothecene Biosynthesis in Fusarium graminearum Species Complex Combined with Transcriptomic and Proteomic Analysis
Source: Microorganisms. 2022 Jul 22;10(8):1479. doi: 10.3390/microorganisms10081479 (PMC9332169; doi:10.3390/microorganisms10081479)

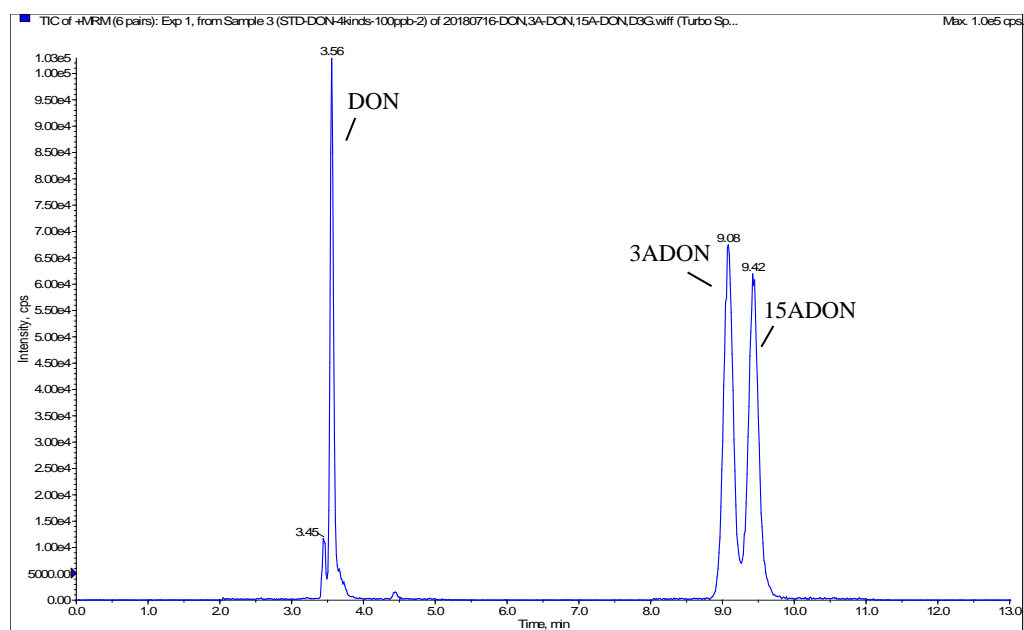

**Figure S1.** Representative LC-MS/MS chromatograms for DON, 3ADON, and 15ADON.

Supplement: Supplementary file 1 [file microorganisms-10-01479-s001.zip › Figure S1.pdf]

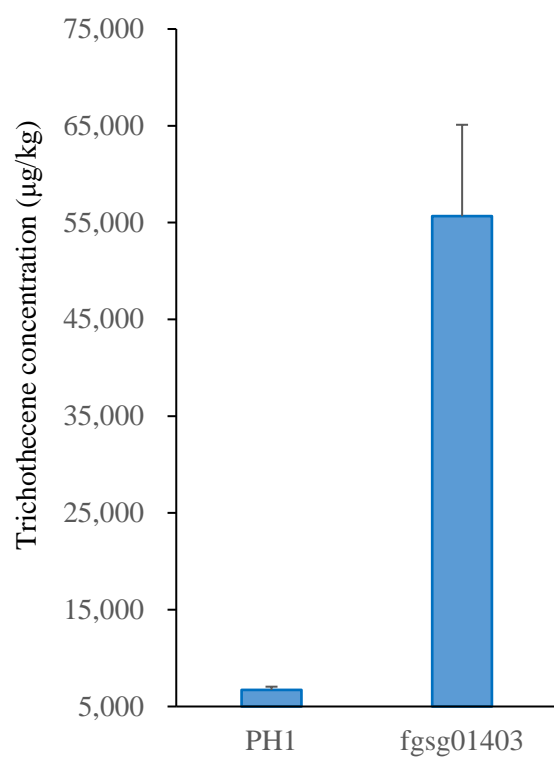

**Figure S4.** Trichothecene production of PH1 and FGSG\_01403 mutant fgsg01403 in 6-day-old rice cultures.

Supplement: Supplementary file 1 [file microorganisms-10-01479-s001.zip › Figure S4.pdf]

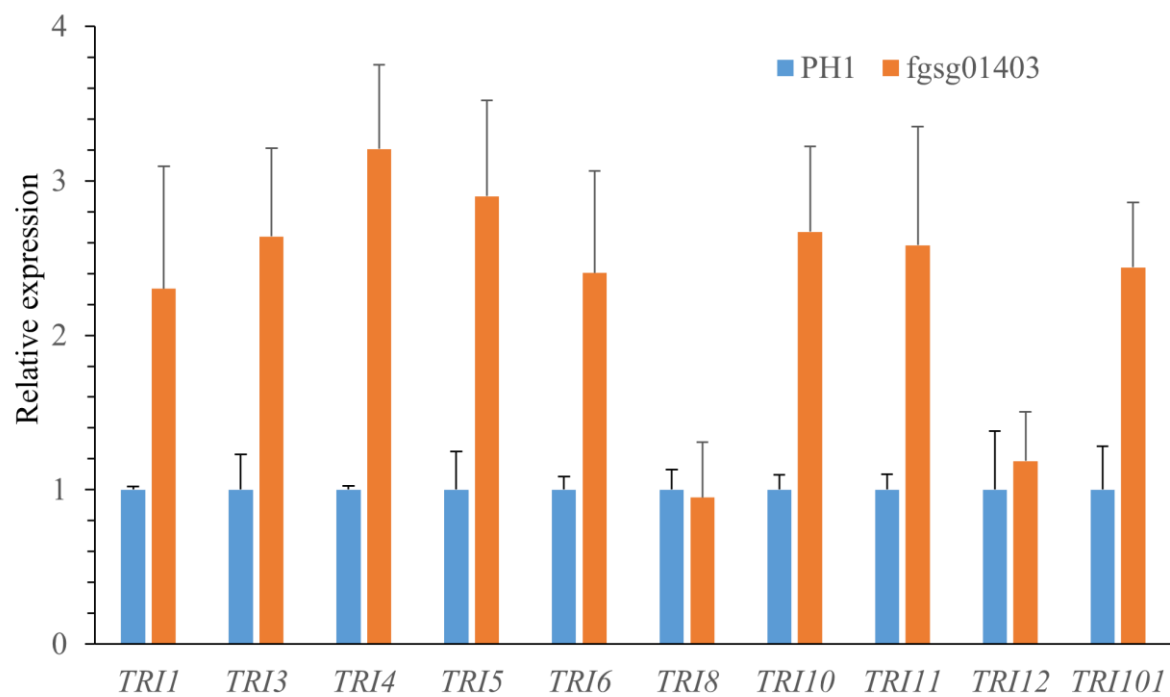

**Figure S5.** The expression levels of *TRI* genes in the wild-type PH1 and *FGSG\_01403* mutant fgsg01403.

Supplement: Supplementary file 1 [file microorganisms-10-01479-s001.zip › Figure S5.pdf]
